# Identification of *TCR Vβ*11-2-*Dβ*1-*Jβ*1-1 T cell clone specific for WT1 peptides using high-throughput *TCRβ* gene sequencing

**DOI:** 10.1186/s40364-019-0163-1

**Published:** 2019-06-14

**Authors:** Yikai Zhang, Ling Xu, Shaohua Chen, Xianfeng Zha, Wei Wei, Yangqiu Li

**Affiliations:** 1 Key Laboratory for Regenerative Medicine of Ministry of Education, Institute of Hematology, School of Medicine, Jinan University, 601 Huang Pu Da Dao Xi, 510632 Guangzhou, People’s Republic of China; 2 Department of Hematology, First Affiliated Hospital, Jinan University, Guangzhou, 510632 China; 3 Department of Clinical Laboratory, First Affiliated Hospital, Jinan University, Guangzhou, 510632 China; 4 Guangzhou Municipality Tianhe Nuoya Bio-engineering Co. Ltd, Guangzhou, 510663 China

**Keywords:** Chronic myelogenous leukemia, Wilms tumor 1, BCR-ABL, T cell repertoire, T-cell receptor beta-chain sequencing

## Abstract

We previously identified a *TCR Vβ*21 T cell clone which was specific to CML patients, and demonstrated that *TCR Vα*13/*β*21 gene-modified CD3^+^ T cells had specific cytotoxicity for HLA-A11^+^ K562 cells. However, it remains unclear which antigen is specifically recognized by the *TCR Vβ*21 T cell clone. In this study, CD3^+^ T cells from healthy donor peripheral blood were stimulated with the WT1 peptide or mixed BCR-ABL peptides in the presence or absence of IL-2 and IL-7. The distribution of the *TCR Vβ* repertoire was analyzed after different stimulations. We found that the mixed BCR-ABL peptides induced clonally expanded *Vβ*7–9-*Dβ*2-*Jβ*2–7 T cells while the Wilms Tumor 1 peptide induced clonally expanded *Vβ*11–2-*Dβ*1-*Jβ*1–1 T cells by high-throughput *TCRβ* sequencing and GeneScan. Interestingly, the sequence and CDR3 motif of *Vβ*11–2 T cell clone are similar to the *TCR Vβ*21 (a different *TCR V* region naming system) T cell clone that we previously found in CML patients. Thus, our findings suggest that the *TCR Vβ*21 T cell clone found in CML patients might be a T cell clone that specifically recognizes WT1.

**Electronic supplementary material:**

The online version of this article (10.1186/s40364-019-0163-1) contains supplementary material, which is available to authorized users.

## Background

Chronic myelogenous leukemia (CML) is a common hematological malignancy in adults and has the molecular characteristic of BCR-ABL fusion proteins, which exhibit abnormal kinase activity [1]. Although many CML patients benefited from the development and application of tyrosine kinase inhibitors (TKIs) [2], a part of patients still suffer from primary and acquired resistance to TKIs [3, 4]. Another therapeutic approach for CML is hematopoietic stem cell transplantation (HSCT), including allogenic-HSCT and haploidentical-HSCT; however, their use is limited for older patients [5, 6]. There is evidence demonstrating that CML patients who have undergone recurrent allo-HSCT could be aided by donor lymphocyte infusion (DLI) [7–9]. These findings suggest that adoptive T cell immunotherapy may be a potentially effective strategy for CML patients.

Mechanistically, infusing donor-derived cytotoxic T lymphocytes (CTLs) induces CTL-mediated leukemia cell death through the recognition of leukemia-associated antigens. However, DLI also causes graft-versus-host disease (GVHD), mainly because CTLs are multi-clonal T cells that also recognize allo-antigens expressed in host-normal tissues [10]. Therefore, infusing leukemic antigen-specific CTLs is a better strategy for overcoming GVHD for adoptive T cell immunotherapy.

Wilms Tumor 1 (WT1) is a tumor suppressor gene involved in the etiology of Wilms’ tumor. It is overexpressed mainly in myeloid leukemias, such as acute myeloid leukemia (AML) and CML, myelodysplastic syndrome (MDS), and several solid tumors [11–14]. There are evidences demonstrating that WT1 overexpression is closely associated with CML progression, and the poor therapeutic effect of TKIs [15–19]. These results highlight WT1 as a common therapeutic target for leukemia. For example, a clinic trail showed that WT1 peptide vaccination in WT1-expressing AML and MDS patients without curative treatment option had clinical benefit including complete remission or stable diseases (SDs) with more than 50% blast reduction. The effect is accompanied by the emergence of a predominant *TCR Vβ +* T cell clone both in blood and bone marrow [20–23]. However, the types of leukemia associated-antigens in AML patients are relatively complex, thus not allowing a clear definition of the types of antigens recognized by clonally expanded *TCR Vβ* T cells after injection with a WT1 vaccine.

We previously identified a TCR *Vβ*21 monoclone in blood from patients with CML and demonstrated that *TCR Vα13/β*21 gene-modified T cells could induce cell death in HLA-A11^+^ K562 cells [24, 25]. However, it remains unclear whether *TCR Vβ*21 T cell clones specifically recognize BCR-ABL or other CML-associated antigens. Therefore, in this study, we analyzed the distribution of the *TCR Vβ* repertoire in CD3^+^ T cells from healthy donor peripheral blood after different stimulations with a WT1 peptide or mixed BCR-ABL peptides in the presence or absence of interleukin (IL)-2 and IL-7.

## Materials and methods

### CD3^+^ T cell sorting

Peripheral blood mononuclear cells (PBMCs) were isolated from the peripheral blood of a healthy HLA-0201^+^ donor with informed consent by Ficoll-Hypaque gradient centrifugation. CD3^+^ T cells were then sorted by immunomagnetic beads from the PBMCs. The immunnomagnetic beads were purchased from MACS, and the sorting operation was performed according to the manufacturer’s instructions (Miltenyi Biotec, Germany). This study was approved in writing by the Ethics Committee of the first affiliated hospital of Jinan University.

### Cell culture and treatment

CD3^+^ T cells (2.5 X 10^6^ cells/mL) were cultured in RPMI 1640 without fetal bovine serum overnight. Fresh media containing 100 UI/mL IL-7, 100 UI/mL IL-2, and antigen peptides was then added to the cells. Untreated cells served as the control group, and the cytokines group comprised cells treated only with IL-2 and IL-7. The cells in the WT1 group were treated with a WT1-specific antigen peptide (RMFPNAPYL HLA A0201), while the cells in the BCR-ABL (B3A2) group were treated with six mixed antigen BCR-ABL peptides (Additional file 1: Table S1). The cells were cultured for 3 weeks. IL-2 was added into the media twice a week, and IL-7 was added into the media once a week. Finally, cultured T cells from different groups were collected for RNA isolation.

### RNA extraction and TCRβ sequencing

Total RNA was extracted from samples with TRIzol (Invitrogen, 15,596) according to the manufacturer’s instructions. The RNA was dissolved by ddH_2_O after drying out. Then, the immune library sequences were amplified by 5′rapid amplification of cDNA ends (RACE). After amplification, the concentration and integrity of the fragments were determined by Qubit, Agilent, and Q-PCR. Qualified libraries were sequenced by HiSeq or MiSeq. The mixcr (v1.8.2) program was used to identify the sequences in each sample. Sequences containing the complementarity-determining region 3 (CDR3) that had greater than four amino acids and a nucleic acid length that was a multiple of three without stop codon were retained as qualified clones. Bioinformatics analysis was performed after obtaining qualified clones. The amplification and sequencing of *TCR Vβ* and primary analysis were performed by the Huayin Health Company.

### RT-PCR, sanger sequencing and GeneScan analysis for TCR Vβ subfamily clonality

Twenty-four *TCR* V*β* primers and a *TCR* C*β* primer were used in unlabeled PCR to amplify the *TCR* V*β* subfamily members. PCR was performed as described in our previous study. A portion of the PCR product was used for direct sequencing, which was performed by Invitrogen Biotechnology Company. The sequences of the different samples were analyzed with BLAST (https://blast.ncbi.nlm.nih.gov/Blast.cgi). The remaining PCR product was used to perform runoff PCR with the addition of fluorescent primers labeled at the 5′ end with a FAM (5-Carboxyfluorescein) fluorophore (C*β*-FAM) (TIB MOLBIOL GmbH, Germany). Then, the labeled runoff PCR products (2.0 μL) were mixed with 9.5 μL formamide (Hi-Di Formamide, ABI, USA) and 0.5 μL Size Standards (GENESCAN™-500-LIZ™, Perkin Elmer, ABI) and heat-denatured at 94 °C for 4 min. The samples were resolved by electrophoresis using a 310 DNA sequenator (Perkin Elmer, ABI) in a 310 POP-4™ gel (Performance Optimized Polymer-4, ABI). The size and fluorescence intensity were determined by GeneScan software. The PCR protocol was performed as described in our previous study [26, 27].

## Results

### Distribution of the TCR Vβ repertoire in T cells after stimulation with WT1 and BCR-ABL peptides

We treated four groups of CD3^+^ T cells from the same healthy donor’s PBMCs under different conditions and then analyzed the distribution of the *TCR Vβ* repertoire by *TCRβ* gene sequencing. Approximately 16.1 million effective reads were generated from the CD3-positive T cell populations (Table 1). There were approximately 60 types of the *Vβ* gene and 14 types of the *Jβ* gene detected in each group. The number of unique VDJ rearrangements was approximately 1.4 thousand in each group. The number of unique CDR3 amino acid sequences (corresponding to an in-frame effective rearrangement of the CDR3 nucleotide sequence) in the control group was 17,789, while it was 13,828 in the cytokines group. For the WT1 and B3A2 groups, the numbers were 14,472 and 11,747, respectively. From these data, we could also determine that there was no significant difference in the numbers of *Vβ* genes, *Jβ* genes, VDJ gene rearrangements, and unique CDR3 amino acid sequences in the four groups.

**Table 1:** Classification and counts for the sequencing results

Sample	Read pairs	Effective reads	Unique *Vβ*	Unique *Jβ*	Unique VDJ	Unique CDR3aa
Control	5227607	4297968	62	14	1499	17789
Cytokines	5110352	3254496	64	14	1448	13828
WT1	6591869	4968087	62	14	1437	14472
B3A2	5854623	3542234	64	14	1393	11747

Control: the untreated group; Cytokines: cells cultured with IL-2 and Il-7 in vitro for 21 days; WT1: cells treated with WT1 peptide (RMFPNAPYL, AA 126–134) and cultured with IL-2 and IL-7 in vitro for 21 days; B3A2: cells treated with 6 different BCR-ABL peptides and cultured with IL-2 and IL-7 in vitro for 21 days; An effective read is a read possessing the full CDR3 structure; Unique *Vβ*: types of *Vβ* gene usage; Unique *Jβ*: types of *Jβ* gene usage; Unique VDJ: unique combinations of V/D/J genes; Unique CDR3aa: unique CDR3 amino acid sequences

**Fig. 1:**
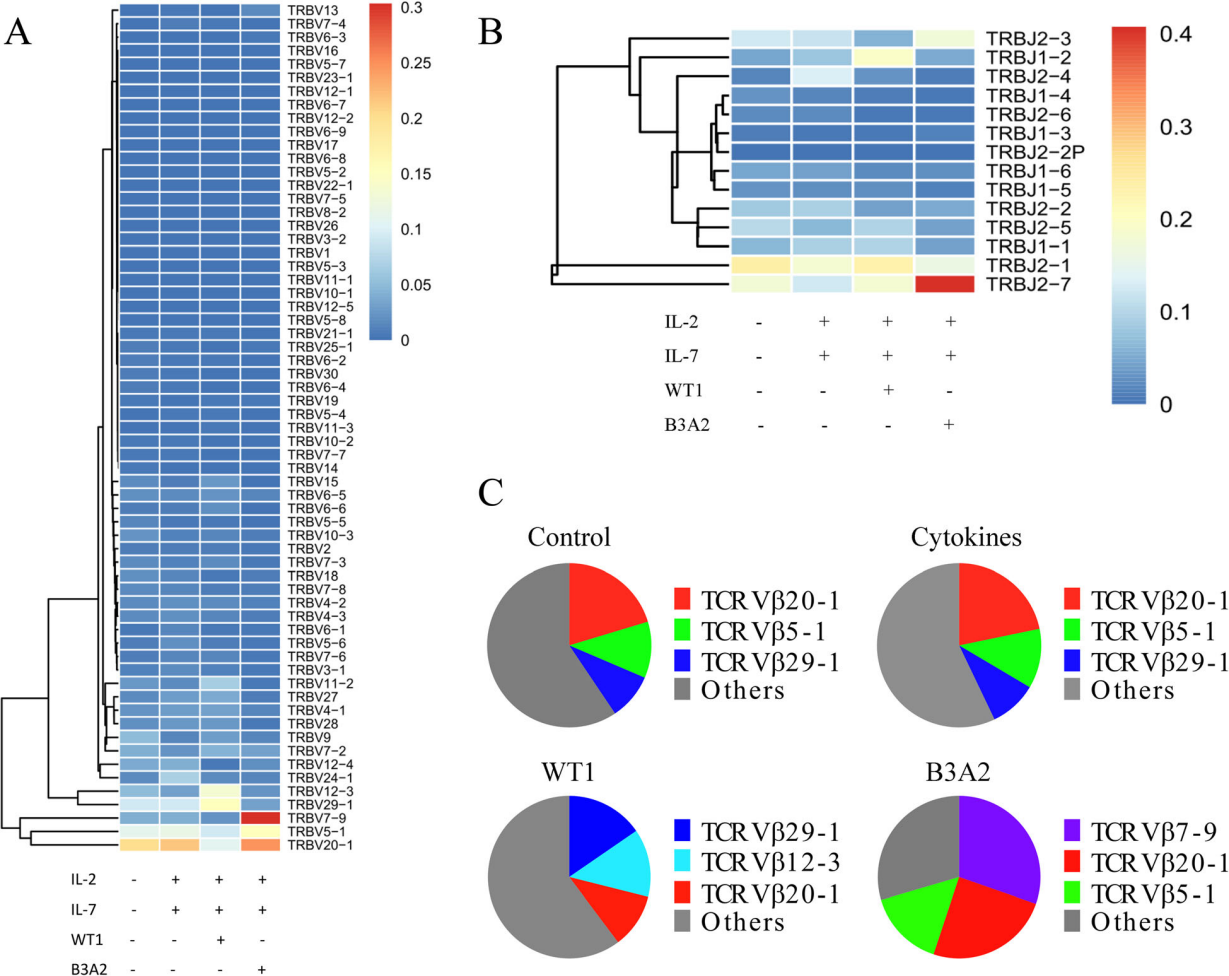
The expression frequency of the *Vβ* and *Jβ* genes in the four groups. **a** The expression frequency of the *Vβ* gene; **b** The expression frequency of the J*β* gene; **c** The ratios for the top three *Vβ* subfamilies in the different groups

However, there were some differences in the usage of the *Vβ* genes and *Jβ* segments among the four groups (Fig. 1a, b). To obtain more detail regarding differences in the four groups, we further analyzed the data by the frequency of the *Vβ* subfamilies. We found that the top three *Vβ* subfamilies in the control group were *Vβ*20–1 (20.3%), *Vβ*5–1 (11.1%), and *Vβ*29–1 (9.2%), which was similar to that found in the cytokines-treated group (unspecific stimulation; Fig. 1c); however, in the WT1 group, the top three *Vβ* subfamilies changed to *Vβ*29–1 (15.4%), *Vβ*12–3 (13.5%), and *Vβ*20–1 (10.8%), while in the B3A2 group, *Vβ*7–9 (30.3%), *Vβ*20–1 (24.8%), and *Vβ*5–1 (15.4%) were the most frequent. These data demonstrate that stimulation with cytokines did not change the type of *Vβ* subfamilies used in normal T cells as expected, while stimulation with different peptides could induce the selective proliferation of different *Vβ* subfamily T cell clones.

### Frequent usage pattern of Vβ VDJ rearrangement in T cells after stimulation with WT1 and BCR-ABL peptides

**Fig. 2:**
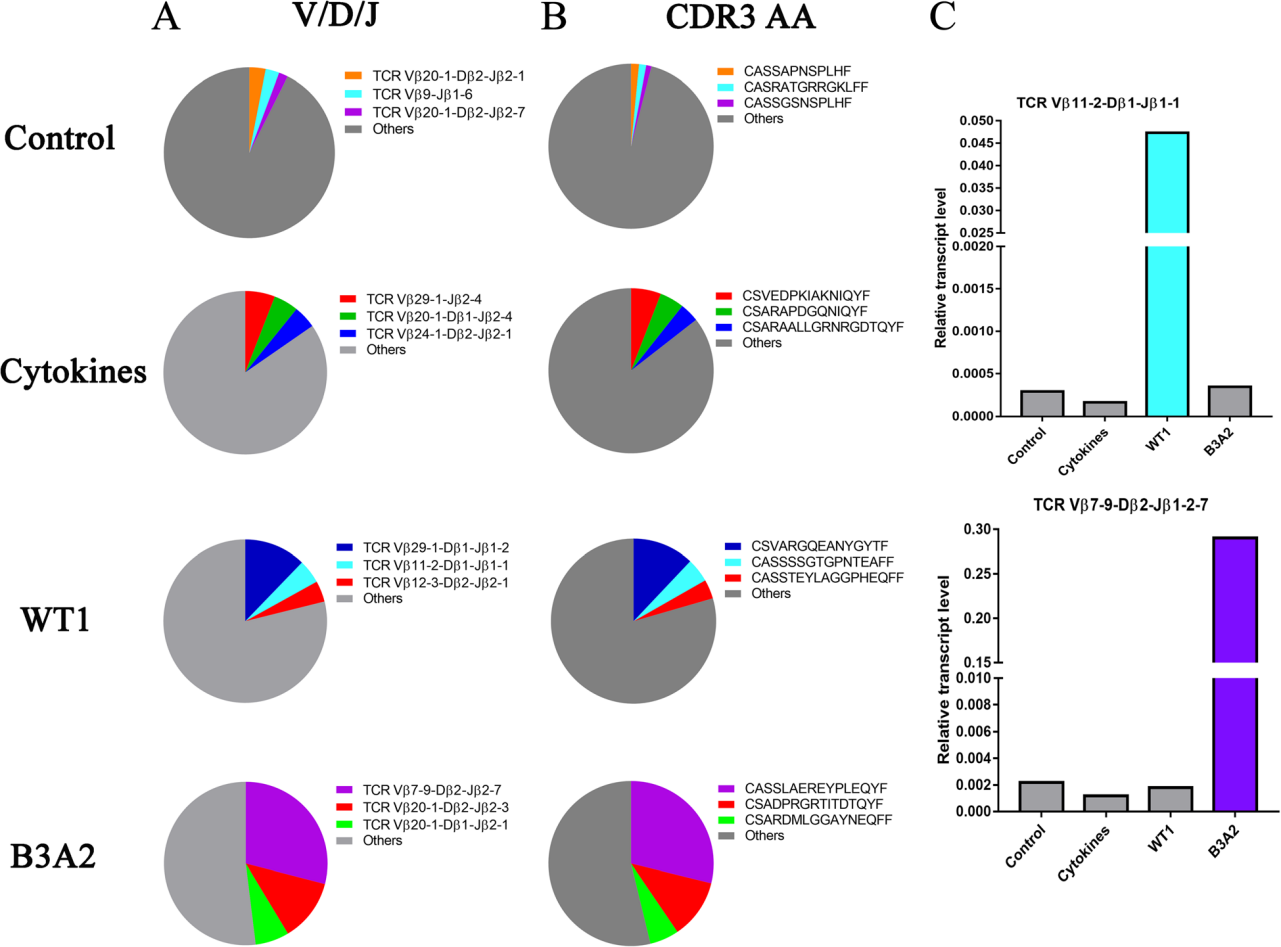
The predominant V (D) J rearrangements and expression levels of the CDR3 amino acids in the four groups. **a** The ratios of predominantly used V (D) J rearrangements in the four groups; **b** The ratios of the CDR3 amino acid in the four groups; **c** The expression levels of the *TCR Vβ*11–2-*Dβ*1-*Jβ*1–1 and *TCR Vβ*7–9-*Dβ*2-*Jβ*2–7 T cell clones in the four groups

It is well known that VDJ recombination is an important event during the proliferation of the T cells, and CDR3 is an antigen recognition region that could be used as a specific marker for each T cell clone. Thus, we analyzed the usage frequency of VDJ recombination and the expression frequency of the predicted CDR3 amino acid sequences in the four groups. As shown in Fig. 2a, the top three VDJ rearrangements in the control group were *Vβ*20-1-*Dβ*2-*Jβ*2-1 (3.1%), *Vβ*9-*Jβ*1-6 (2.6%), and *Vβ*20-1-*Dβ*2-*Jβ*2-7 (1.7%), while in the cytokines group, it was *TCR Vβ*29-1-*Jβ*2-4 (5.9%), *Vβ*20-1-*Dβ*1-*Jβ*2-4 (4.9%), and *Vβ*24-1-*Dβ*2-*Jβ*2-1 (4.5%). The VDJ rearrangement usage pattern changed in the WT1 and B3A2 groups. The top three VDJ rearrangements in the WT1 group were *Vβ*29-1-*Dβ*1-*Jβ*1-2 (12.2%), *Vβ*11-2-*Dβ*1-*Jβ*1-1 (4.7%), and *V*12-3-*Dβ*2-*Jβ*2-1 (4.2%). In the B3A2 group, they were *Vβ*7-9-*Dβ*2-*Jβ*2-7 (29.0%), *Vβ*20-1-*Dβ*2-*Jβ*2-3 (12.4%), and *Vβ*20-1-*Dβ*1-*Jβ*2-1 (6.7%). Similarly, the expression types and frequencies of the CDR3 amino acid sequences were different in the four groups (Fig. 2b). We then analyzed the frequencies of the VDJ rearrangements corresponding to the CDR3 amino acids in the four groups. We found that the VDJ rearrangements corresponding to the highly expressing CDR3 amino acids were also the patterns most highly used in each group. The highest expressed CDR3 amino acid sequence (CASSLAEREYPLEQYF; 28.8%) in the B3A2 group was identified as a *TCR Vβ*7-9-*Dβ*2-*Jβ*2-7 rearrangement, while the second highest expressed CDR3 amino acid sequence (CASSSSGTGPNTEAFF) (4.7%) in the WT1 group was identified as a *TCR Vβ*11-2-*Dβ*1-*Jβ*1-1 rearrangement (Fig. 2c). Interestingly, this T cell clone was similar to the clonally expanded *Vβ*21 (different *TCR V* region naming system) in T cells from CML patients that we identified previously.

### Identification of a WT1- or BCR-ABL-specific TCR Vβ clone

**Fig. 3:**
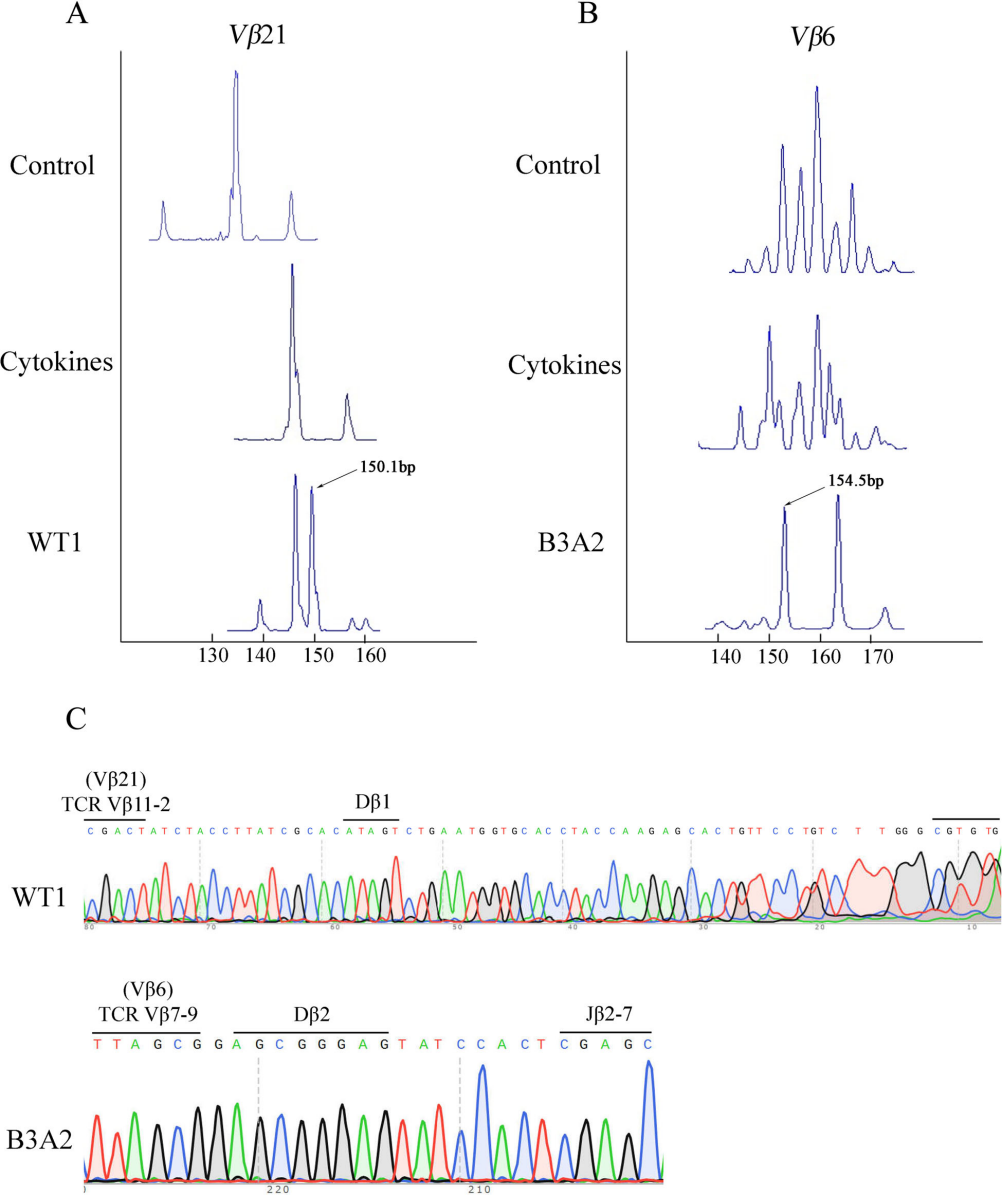
GeneScan and Sanger sequencing results. **a** GeneScan results from *Vβ*21-amplified products in the WT1 group. **b** GeneScan results from the *Vβ*6-amplified products in the B3A2 group. **c** The VDJ rearrangement of the *TCR Vβ*11–2-*Dβ*1-*Jβ*1–1 and *TCR Vβ*7–9-*Dβ*2-*Jβ*2–7 T cell clones

We employed RT-PCR and GeneScan for further analysis. Based on GeneScan analysis (Fig. 3a), we found that the *Vβ*21 clone in the WT1 group was biclonal (bi-peaks) and the size of the fragment was approximately 150 bp. In the B3A2 group (Fig. 3b), we found that the *Vβ*6 clone was biclonal, and the control and cytokines groups were polyclonal (multi-peaks). The sequencing results (Fig. 3c) indicated that the CDR3 sequence of *TCR Vβ*6 in the B3A2 group was from the *TCR Vβ*7-9-*Dβ*2-*Jβ*2-7 clone, and the CDR3 sequence of *TCR Vβ* 21 in the WT1 group was from the *TCR Vβ*11-2-*Dβ*1-*Jβ*1-1 clone.

## Discussion

Adoptive T cell transfusion using cancer antigen-specific T cells is the most effective immunotherapy [28]. However, there are issues that limit the application of this approach including difficulties in generating a sufficient number of cancer antigen-specific T cells for each patient in vitro in a short period of time, and patient antigen-specific T cells demonstrating low activation [29]. With the exception of chimeric antigene receptor (CAR)-T cells, TCR-engineered T cells have emerged from pre-clinical research to clinical trials and can overcome the low numbers of patient-derived CTLs [30–35]. Thus, identification of tumor antigen-specific TCRs is a key issue for such TCR-T cell generation and application.

To design specific T cell immunotherapies for CML, the identification of common CML-specific TCRs might be focused on BCR-ABL or WT1 antigens [36–39]. Based on our previous finding that the *TCR Vα1*3/*β*21 gene derived from CML patient-modified CD3^+^ T cells can specifically target HLA-A11^+^ K562 cells [25], it would be interesting to further characterize the target of this TCR.

In this study, we first compared the frequent usage of the *TCR Vβ* repertoire in CD3^+^ T cells treated with WT1 or BCR-ABL peptides by high-throughput *TCR*β gene sequencing. The predominant *TCR Vβ* clone in WT1 peptide-induced T cells was *TCR Vβ*11-2, while *TCR Vβ*7-9 was predominant in BCR-ABL mixed peptide-induced T cells. As expected, the clonal response of the *TCRV*β subfamily cells appeared to vary with different leukemia-associated antigen epitopes. To confirm this finding, we detected the expression of both *Vβ*11-2 and *Vβ*7-9 in treated CD3^+^ T cell samples by RT-PCR, and the PCR products were further analyzed by GeneScan to confirm the clonality of the T cells and direct Sanger sequencing to confirm the CDR3 rearrangement [40]. Significantly, clonal expansion of T cells expressing *TCR Vβ*11-2 or *TCR Vβ*7-9 was identified, and the CDR3 sequences were also confirmed as *Vβ*11-2-*Dβ*1-*Jβ*1-1 and *Vβ*7-9-*Dβ*2-*Jβ*2-7, respectively. Both are in-frame rearrangements, and their predicted CDR3 amino acid sequences were CASSSSGTGPNTEAFF (WT1 related) and CASSLAEREYPLEQYF (BCR-ABL related). Therefore, both novel TCR clones might be responsible for BCR-ABL or WT1 epitopes, and whether they could be used to produce TCR-modified T cells requires further investigation. Interestingly, we found that the *TCR* V*β*11-2-*Dβ*1-*Jβ*1-1 sequence is similar to the sequence in the *Vβ*21 T cell clone (a different *TCR V* region naming system) in CML patients that we previously identified, which could mediate specific cytotoxicity against CML with the TCR-modified T cell technique [25]. Whether these *Vβ*21 T cell clones from CML patients specifically recognize the WT1 peptide or cross respond based on different individuals requires more investigation. Previous studies have been showed that WT1 specific T cell clones were induced in AML patients by the same WT1_126–134_ peptide vaccines which was used in this study, such T cell clone also expressed *TCR Vβ*11, however, the CDR3 sequence (ASSDYNEQF) is different from the *TCR* V*β*11-2-*Dβ*1-*Jβ*1-1 (CASSSSGTGPNTEAFF) which we found in the study and the CML patients [20, 41–43]. The reason that different TCR clone amplification induced by the same peptide, is due to the individual T cell response from different donors and patients. There were also studies showing different TCR clone (*TCR Vβ*5-1-*Dβ*2-*Jβ*2-5) inducted by WT1 (CMTWNQMNL) peptides [44]. Overall, the new identified *TCR* V*β*11-2-*Dβ*1-*Jβ*1-1 clone in this study may provide new data of WT1 specific TCR clone bank. Moreover, on the other hand, all of this identified TCR clone may be thought as one of the immune biomarker of WT1 specific T cell clone in WT1 + malignancies.

## Conclusion

In summary, we characterized the different usage patterns of the *TCRVβ* repertoire in T cells after WT1 and BCR-ABL peptide stimulation and identified two novel TCR clones (*Vβ*11-2-*Dβ*1*-Jβ*1-1 and *Vβ*7-9-*Dβ*2-*Jβ*2-7) related to both antigens. Functional studies will be performed to confirm their anti-CML cytotoxicity by producing TCR gene-modified T cells, it may be possible to provide a new TCR-T cell clone for WT1 + leukemia and maybe for WT1 + solid tumors immunotherapy.

## Additional file

Additional file 1: **Table S1.** Sequences of the BCR-ABL antigen peptides. (DOCX 17 kb)

## Data Availability

The datasets used and/or analyzed during the current study are available from the corresponding author on reasonable request.

## Abbreviations

AML: Acute myeloid leukemia
CAR: Chimeric Ag receptor
CDR3: Complementarity-determining region 3
CML: Chronic myelogenous leukemia
CTLs: Cytotoxic T lymphocytes
DLI: Donor lymphocyte infusion
GVHD: Graft-versus-host disease
HSCT: Hematopoietic stem cell transplantation
IL: Interleukin
MDS: Myelodysplastic syndrome
PBMCs: Peripheral blood mononuclear cells
RACE: Rapid amplification of cDNA ends
SDs: Stable diseases
TKIs: Tyrosine kinase inhibitors
WT1: Wilms Tumor 1

## References

[1] Chopra R, Pu QQ, Elefanty AG (1999). Biology of BCR-ABL. Blood Rev.

[2] Druker BJ (2001). Activity of a specific inhibitor of the BCR-ABL tyrosine kinase in the blast crisis of chronic myeloid leukemia and acute lymphoblastic leukemia with the Philadelphia chromosome (vol 344, pg 1038, 2001). N Engl J Med.

[3] Jangamreddy JR, Panigrahi S, Lotfi K, Yadav M, Maddika S, Tripathi AK (2014). Mapping of Apoptin-interaction with BCR-ABL1, and development of apoptin-based targeted therapy. Oncotarget.

[4] Marin D, Milojkovic D, Olavarria E, Khorashad JS, de Lavallade H, Reid AG (2008). European LeukemiaNet criteria for failure or suboptimal response reliably identify patients with CML in early chronic phase treated with imatinib whose eventual outcome is poor. Blood.

[5] Hehlmann R, Hochhous A, Baccarani M, European L (2007). Chronic myeloid leukaemia. Lancet.

[6] Xu L, Chen H, Chen J, Han M, Huang H, Lai Y (2018). The consensus on indications, conditioning regimen, and donor selection of allogeneic hematopoietic cell transplantation for hematological diseases in China-recommendations from the Chinese Society of Hematology. J Hematol Oncol.

[7] Collins RH, Shpilberg O, Drobyski WR, Porter DL, Giralt S, Champlin R (1997). Donor leukocyte infusions in 140 patients with relapsed malignancy after allogeneic bone marrow transplantation. J Clin Oncol.

[8] Kolb HJ, Schmid C, Barrett AJ, Schendel DJ (2004). Graft-versus-leukemia reactions in allogeneic chimeras. Blood.

[9] Pinilla-Ibarz J, Shah B, Dubovsky JA (2009). The biological basis for immunotherapy in patients with chronic myelogenous leukemia. Cancer Control.

[10] Dazzi F, Goldman J (1999). Donor lymphocyte infusions. Curr Opin Hematol.

[11] Rosenfeld C, Cheever MA, Gaiger A (2003). WT1 in acute leukemia, chronic myelogenous leukemia and myelodysplastic syndrome: therapeutic potential of WT1 targeted therapies. Leukemia.

[12] Yang L, Han Y, Saiz FS, Minden MD (2007). A tumor suppressor and oncogene: the WT1 story. Leukemia.

[13] Sugiyama H (2010). WT1 (Wilms’ tumor gene 1): biology and Cancer immunotherapy. Jpn J Clin Oncol.

[14] Yoon JH, Kim HJ, Kwak DH, Park SS, Jeon YW, Lee SE (2017). High WT1 expression is an early predictor for relapse in patients with acute promyelocytic leukemia in first remission with negative PML-RARa after anthracycline-based chemotherapy: a single-center cohort study. J Hematol Oncol.

[15] Cilloni D, Messa F, Gottardi E, Fava M, Arruga F, Defilippi L (2004). Sensitivity to imatinib therapy may be predicted by testing Wilms tumor gene expression and colony growth after a short in vitro incubation. Cancer.

[16] Svensson E, Vidovic K, Lassen C, Richter J, Olofsson T, Fioretos T (2007). Deregulation of the Wilms’ tumour gene 1 protein (WT1) by BCR/ABL1 mediates resistance to imatinib in human leukaemia cells. Leukemia.

[17] Otahalova E, Ullmannova-Benson V, Klamova H, Haskovec C (2009). WT1 expression in peripheral leukocytes of patients with chronic myeloid leukemia serves for the prediction of Imatinib resistance. Neoplasma.

[18] Vidovic K, Svensson E, Nilsson B, Thuresson B, Olofsson T, Lennartsson A (2010). Wilms’ tumor gene 1 protein represses the expression of the tumor suppressor interferon regulatory factor 8 in human hematopoietic progenitors and in leukemic cells. Leukemia.

[19] Szanto A, Pap Z, Denes L, Lazar EB, Horvath A, Tunyogi AB (2015). Real-time quantitative PCR detection of WT1 and M-BCR-ABL expressions in chronic myeloid leukemia. Romanian J Morphol Embryol.

[20] Keilholz U, Letsch A, Busse A, Asemissen AM, Bauer S, Blau IW (2009). A clinical and immunologic phase 2 trial of Wilms tumor gene product 1 (WT1) peptide vaccination in patients with AML and MDS. Blood.

[21] Ochsenreither S, Fusi A, Busse A, Bauer S, Scheibenbogen C, Stather D (2011). “Wilms tumor protein 1” (WT1) peptide vaccination-induced complete remission in a patient with acute myeloid leukemia is accompanied by the emergence of a predominant T-cell clone both in blood and bone marrow. J Immunother.

[22] Ochsenreither S, Fusi A, Geikowski A, Stather D, Busse A, Stroux A (2012). Wilms’ tumor protein 1 (WT1) peptide vaccination in AML patients: predominant TCR CDR3beta sequence associated with remission in one patient is detectable in other vaccinated patients. Cancer Immunol Immunother.

[23] Liu HT, Zha YY, Choudhury N, Malnassy G, Fulton N, Green M (2018). WT1 peptide vaccine in Montanide in contrast to poly ICLC, is able to induce WT1-specific immune response with TCR clonal enrichment in myeloid leukemia. Exp Hematol Oncol.

[24] Zha X, Chen S, Yang L, Li B, Chen Y, Yan X (2011). Characterization of the CDR3 structure of the Vbeta21 T cell clone in patients with P210(BCR-ABL)-positive chronic myeloid leukemia and B-cell acute lymphoblastic leukemia. Hum Immunol.

[25] Zha XF, Xu L, Chen SH, Yang LJ, Zhang YK, Lu YH (2016). Generation of V alpha 13/beta 21(+)T cell specific target CML cells by TCR gene transfer. Oncotarget.

[26] Jin ZY, Wu XL, Chen SH, Yang LJ, Liu QF, Li YQ (2014). Distribution and Clonality of the V alpha and V beta T-cell receptor repertoire of regulatory T cells in leukemia patients with and without graft versus host disease. DNA Cell Biol.

[27] Li YQ, Geng SX, Du X, Chen SH, Yang LJ, Wu XL (2011). Restricted TRBV repertoire in CD4(+) and CD8(+) T-cell subsets from CML patients. Hematology.

[28] Dossa RG, Cunningham T, Sommermeyer D, Medina-Rodriguez I, Biernacki MA, Foster K (2018). Development of T-cell immunotherapy for hematopoietic stem cell transplantation recipients at risk of leukemia relapse. Blood.

[29] Sandri S, Bobisse S, Moxley K, Lamolinara A, De Sanctis F, Boschi F (2016). Feasibility of telomerase-specific adoptive T-cell therapy for B-cell chronic lymphocytic leukemia and solid malignancies. Cancer Res.

[30] Ochi Toshiki, Fujiwara Hiroshi, Yasukawa Masaki (2010). Application of Adoptive T-Cell Therapy Using Tumor Antigen-Specific T-Cell Receptor Gene Transfer for the Treatment of Human Leukemia. Journal of Biomedicine and Biotechnology.

[31] Im A, Pavletic SZ (2017). Immunotherapy in hematologic malignancies: past, present, and future. J Hematol Oncol.

[32] Tawara I, Kageyama S, Miyahara Y, Fujiwara H, Nishida T, Akatsuka Y (2017). Safety and persistence of WT1-specific T-cell receptor gene-transduced lymphocytes in patients with AML and MDS. Blood.

[33] Yu SN, Li AP, Liu Q, Li TF, Yuan X, Han XW (2017). Chimeric antigen receptor T cells: a novel therapy for solid tumors. J Hematol Oncol.

[34] Li ZH, Song WR, Rubinstein M, Liu DL (2018). Recent updates in cancer immunotherapy: a comprehensive review and perspective of the 2018 China Cancer immunotherapy workshop in Beijing. J Hematol Oncol.

[35] Zhang YK, Li YQ (2019). T cell receptor-engineered T cells for leukemia immunotherapy. Cancer Cell Int.

[36] Yamagami T, Sugiyama H, Inoue K, Ogawa H, Tatekawa T, Hirata M (1996). Growth inhibition of human leukemic cells by WT1 (Wilms tumor gene) antisense oligodeoxynucleotides: implications for the involvement of WT1 in leukemogenesis. Blood.

[37] Anuchapreeda S, Thanarattanakorn P, Sittipreechacharn S, Chanarat P, Limtrakul P (2006). Curcumin inhibits WT1 gene expression in human leukemic K562 cells. Acta Pharmacol Sin.

[38] Kerst G, Bergold N, Viebahn S, Gieseke F, Kalinova M, Trka J (2008). WT1 protein expression in slowly proliferating myeloid leukemic cell lines is scarce throughout the cell cycle with a minimum in G (0)/G (1) phase. Leuk Res.

[39] Semsri S, Krig SR, Kotelawala L, Sweeney CA, Anuchapreeda S (2011). Inhibitory mechanism of pure curcumin on Wilms’ tumor 1 (WT1) gene expression through the PKCalpha signaling pathway in leukemic K562 cells. FEBS Lett.

[40] Chen SH, Huang X, Zheng HT, Geng SX, Wu XL, Yang LJ (2013). The evolution of malignant and reactive gamma delta plus T cell clones in a relapse T-ALL case after allogeneic stem cell transplantation. Mol Cancer.

[41] Mailander V, Scheibenbogen C, Thiel E, Letsch A, Blau IW, Keilholz U (2004). Complete remission in a patient with recurrent acute myeloid leukemia induced by vaccination with WT1 peptide in the absence of hematological or renal toxicity. Leukemia.

[42] Zhao Q, Ahmed M, Tassev DV, Hasan A, Kuo TY, Guo HF (2015). Affinity maturation of T-cell receptor-like antibodies for Wilms tumor 1 peptide greatly enhances therapeutic potential. Leukemia.

[43] Nguyen THO, Tan ACL, Xiang SD, Goubier A, Harland KL, Clemens EB (2017). Understanding CD8(+) T-cell responses toward the native and alternate HLA-A*02:01-restricted WT1 epitope. Clin Transl Immunology.

[44] Watanabe K, Toji S, Ohtake J, Nakano K, Satoh T, Kitamura H (2013). Establishment of a stable T lymphoma cell line transduced with HLA-A*24:02-restricted WT1-specific TCR genes and its application to antigen-specific immunomonitoring. Biomed Res Tokyo.

