# Supplementary material for: Identification of TCR Vβ11-2-Dβ1-Jβ1-1 T cell clone specific for WT1 peptides using high-throughput TCRβ gene sequencing
Source: Biomark Res. 2019 Jun 14;7:12. doi: 10.1186/s40364-019-0163-1 (PMC6570921; doi:10.1186/s40364-019-0163-1)
Supplement: Supplementary file 1 — Table S1. Sequences of the BCR-ABL antigen peptides. (DOCX 17 kb) [file 40364_2019_163_MOESM1_ESM.docx]

**Supplementary Table 1. Sequences of the BCR-ABL antigen peptides.**

| Peptide | Sequence | Epitope Source | HLA Allele Restriction |
| --- | --- | --- | --- |
| 1 | GVRGRVEEI | bcr/c-abl | A*02:01 |
| 2 | SSKALQRPV | bcr/c-abl _926-934_ | A*02:01 |
| 3 | KQSSKALQR | bcr/c-abl _924-932_ | A*03:01 |
| 4 | KQSSKALQRPV | bcr/c-abl _924-934_ | A*03:01 |
| 5 | ATGFKQSSK | bcr/c-abl _920-928_ | A*11:01 |
| 6 | GFKQSSKAL | bcr/c-abl _922-930_ | B*08:01 |
